# Nintendo Switch–Based Exergaming for Subthreshold Depression: Mixed Methods Randomized Controlled Trial

**DOI:** 10.2196/80937

**Published:** 2026-06-05

**Authors:** Kexin Huang, Lei Song, Ariadna Albajara Sáenz, Rendong He, Yongliang Jiao, Yong Jia, Li Chen

**Affiliations:** 1 School of Nursing, Jilin University, No.965 Xinjiang Street, Changchun, 130012, China, +86 13194316883; 2 Invasive Technology Nursing Platform, First Hospital of Jilin University, Changchun, China; 3 Department of Psychiatry, University of Cambridge, Cambridge, United Kingdom; 4 Jilin Sport University, Changchun, China

**Keywords:** exergaming, subthreshold depression, Nintendo Ring Fit Adventure, mixed methods design, randomized controlled trial

## Abstract

**Background:**

Subthreshold depression (StD) increases the risk of progression to major depressive disorder. Although exercise can reduce depressive symptoms, adherence remains challenging. Exergames on platforms such as Nintendo Switch may improve motivation and participation in physical activity; however, evidence for efficacy in StD is limited.

**Objective:**

This study aims to evaluate the effects of a Nintendo Switch–based exergaming intervention on depressive symptoms, anxiety, and sleep quality, and to explore participant experiences among adults with StD.

**Methods:**

This sequential explanatory mixed methods study comprised a randomized controlled trial followed by semistructured interviews. Eighty-four adults with StD were randomized using computer-generated permuted blocks with concealed allocation to an intervention group (IG; n=42), which received an 8-week Nintendo Switch–based exergame program (2‐3 sessions/week, 50‐60 minutes/session), or a control group (CG; n=42), which continued usual activities. Outcome assessors and data analysts were blinded. Depressive symptoms, anxiety, and sleep quality were assessed at baseline (T0), postintervention (T1, Week 8), one-month follow-up (T2, Week 12), and 2-month follow-up (T3, Week 16). Generalized estimating equations (GEE) were used to analyze longitudinal changes and time×group interactions under the intention-to-treat principle. Semistructured interviews were conducted with a purposive subsample of IG participants (n=17) at T1 and analyzed using thematic analysis.

**Results:**

Eighty-four participants were randomized; 81 completed the postintervention assessment, with 3 out of 42 (7.1%) CG participants lost to follow-up at T1. Baseline characteristics were similar across groups (mean age 23.07, SD 1.45 years; 70/84, 83.3% female). Compared with CG, the IG showed significantly greater reductions in depressive symptoms at all time points (T1: *β*=−4.07, 95% CI −5.84 to −2.30; *P*<.001; T2: *β*=−4.29, 95% CI −6.14 to −2.43; *P*<.001; T3: *β*=−3.81, 95% CI −5.54 to −2.08; *P*<.001), along with significant improvements in sleep quality (T1: *β*=−2.98, 95% CI −4.55 to −1.40; *P*<.001; T2: *β*=−2.19, 95% CI −3.58 to −0.80; *P*=.002; T3: *β*=−2.45, 95% CI −3.81 to −1.09; *P*<.001). Anxiety also improved significantly at T1 (*β*=−2.60, 95% CI −4.70 to −0.50; *P*=.02) and T3 (*β*=−2.38, 95% CI −4.62 to −0.14; *P*=.04). Group×time interactions were significant for depressive symptoms (Wald *χ*^2^_3_=28.18; *P*=.001) and sleep quality (Wald *χ*^2^_3_=23.21; *P*<.001), confirming sustained intervention effects. Qualitative findings supported these results, highlighting immersive engagement, perceived psychophysiological benefits, and adherence facilitators. No adverse events were reported.

**Conclusions:**

A Nintendo Switch–based exergaming intervention was associated with improvements in depressive symptoms, anxiety, and sleep quality in adults with StD. Using mixed methods design, this study provides evidence integrating effectiveness and participant experience, extending prior research focused on other populations or quantitative outcomes. These findings suggest that commercially available exergaming platforms may serve as accessible, engaging tools for early mental health support in real-world settings.

## Introduction

### Background and Rationale

Subthreshold depression (StD) is considered a precursor stage of major depressive disorder (MDD) and represents an important target for early intervention [1]. It is typically characterized by the presence of 2-4 depressive symptoms persisting for at least 2 weeks without meeting the full diagnostic criteria for MDD [2]. Epidemiological studies suggest that StD is highly prevalent in the general population, with estimated rates ranging from 7.3% to 17.2% among adults, and is associated with a significantly increased risk of developing MDD and other psychiatric disorders [3]. In addition, individuals with StD may experience impaired functioning and an elevated risk of suicidal ideation [4]. Therefore, early and effective interventions targeting StD are essential to prevent the progression to MDD and to reduce associated public health burdens.

Exercise therapy is a proven nonpharmacological intervention for reducing depressive symptoms, offering advantages such as low cost, few side effects, and rapid efficacy [5]. However, its repetitive and unengaging nature often limits motivation and long-term adherence. Given that depression is characterized by low mood and diminished interest in activities [6], interventions that incorporate behavioral activation and intrinsic motivation are particularly suitable.

In recent years, serious games, digital games designed for purposes beyond entertainment, such as education, rehabilitation, or health promotion, have increasingly been explored as innovative tools for behavioral and mental health interventions [7]. Within this broader category, exergames represent a specific type of serious game that combines physical activity with interactive gameplay and may help overcome some of the limitations of traditional exercise interventions [8]. During exergaming activities, participants are more likely to experience a state of flow, which has been associated with greater enjoyment and sustained engagement in physical activity [9,10].

Through game mechanics such as goal-oriented tasks, real-time feedback, and reward structures, exergames have emerged as a promising approach for enhancing enjoyment, intrinsic motivation, and adherence to physical activity programs while simultaneously delivering therapeutic benefits. Previous studies have shown that exergames not only facilitate light-to-moderate intensity exercise but also enhance users’ self-efficacy and willingness to engage in physical activity [11], thereby improving adherence [12]. These features may be particularly relevant for individuals with StD, who often exhibit reduced behavioral activation and altered reward sensitivity [13].

Nintendo Switch, featuring somatosensory controls and immersive design, has shown high safety, feasibility, and user satisfaction across health contexts [14,15], and is increasingly applied in neurorehabilitation and mental health care. Studies have shown that Nintendo Switch–based exergames improve physical [16,17] and psychological outcomes across populations [18-21]. Furthermore, the qualitative studies have also shown their feasibility and acceptability among stroke survivors [22] and older adults [23], who reported positive experiences and social enjoyment.

Despite the growing body of research on exergaming interventions, most studies have primarily focused on older adults [18,19] or specific clinical populations [20,21]. Evidence specifically targeting adults with StD remains limited. Moreover, most existing studies have primarily focused on quantitative outcomes, while participants’ subjective experiences and perceptions of exergame-based interventions remain underexplored. Understanding both the effectiveness and user experience of such interventions may provide important insights for optimizing digital mental health strategies.

### Objectives

Therefore, this study, conducted based on a previously published protocol [24], was designed as a mixed methods exergaming effectiveness trial to evaluate a Nintendo Switch–based exergaming intervention using Ring Fit Adventure (RFA; Nintendo Co Ltd) as the intervention platform. The study aimed to: (1) evaluate the effectiveness of a Nintendo Switch–based exergaming intervention in improving depressive symptoms, anxiety symptoms, and sleep quality among adults with StD; and (2) explore participants’ subjective experiences of the intervention, including engagement, perceived benefits, and acceptability.

## Methods

### Trial Design

This mixed methods sequential explanatory design comprised an assessor-blinded, parallel-group randomized controlled trial (RCT) and semistructured qualitative interviews to evaluate the effectiveness of Nintendo Switch–based exergaming in adults with StD. This RCT adhered to the CONSORT (Consolidated Standards of Reporting Trials) 2025 statement (Checklist 1) and the CONSORT-EHEALTH (Consolidated Standards of Reporting Trials of Electronic and Mobile Health Applications and Online Telehealth) extension for electronic and mobile health interventions to enhance transparency and reproducibility of reporting (Checklist 2) [25,26]. The qualitative component followed the APA Journal Article Reporting Standards for Qualitative Research (JARS-Qual; Checklist 3) [27].

### Involvement

No patient or public involvement was incorporated into the design, conduct, reporting, or dissemination of this research.

### Changes to Trial Protocol

The protocol registered in the trial registry differed from the final study protocol in several aspects. First, the sample size was provisionally estimated as 50 participants in total (25 per group) based on previous studies at the time of trial registration. Prior to participant recruitment, a meta-analysis of available studies on exergame-based exercise training for depressive symptoms in adults was conducted to obtain a more precise estimate of the expected effect size [28]. Based on the pooled effect size (0.69), the required sample size was recalculated, resulting in a revised target of 38 participants per group. Second, in the trial registry, StD was defined by the Center for Epidemiologic Studies Depression Scale (CES-D) ≥16 and a 24-item Hamilton Depression Rating Scale (HAMD-24) score between 8 and 20. Prior to recruitment, this criterion was refined to align with contemporary diagnostic practice. In the final protocol, StD was defined as CES-D≥16 in individuals not meeting *DSM-5* (*Diagnostic and Statistical Manual of Mental Disorders, Fifth Edition)* diagnostic criteria for MDD. Third, the trial registry included several additional secondary outcomes (eg, subjective experience, flow experience, physical training, sports motivation, exercise intensity, and BMI). The present report focuses on the primary outcome (depressive symptoms) and 2 secondary outcomes (anxiety symptoms and sleep quality), while the remaining registered outcomes will be reported separately.

### Quantitative Phase: RCT

#### Setting and Participants

Participants were recruited from 3 universities in Northeast China using a combination of strategies, including posters, flyers, social media outreach, and on-site information sessions from January to February 2023. Screening and evaluation were conducted via the WeChat (Tencent)-based Questionnaire Star platform and supplemented with face-to-face assessments. The sample size calculation is described in detail in the previously published study protocol [24], which determined a required sample size of 76 participants (38 per arm).

The participants were included in the study if they met the following criteria: (1) aged 18 years or older; (2) fulfillment of the diagnostic criteria for StD, defined as not meeting the *DSM-5 *criteria for MDD but presenting with a CES-D score of 16 or higher [29]; (3) volunteered and agreed to participate in the study and signed informed consent; and (4) individuals who had not received treatment for depression in the past 6 months and did not plan to receive treatment outside the study during the trial period. Participants were excluded if they: (1) presented an intellectual disability and/or physical limitations (eg, severe mobility, visual, or hearing impairments); (2) had previously participated in the Nintendo Switch–based exergame within the past 6 months; (3) presented other types of mental health disorders; and (4) presented a suicide risk.

#### Intervention

The intervention was delivered by 5 trained facilitators, including 3 registered nurses with clinical experience and 2 faculty members with expertise in psychological nursing and sports medicine. To ensure fidelity, all facilitators completed 2 standardized training sessions covering the study protocol, system operation, safety monitoring, and communication strategies, with practical simulations to standardize delivery. Moreover, participants also received 2 60-minute structured preintervention training sessions from facilitators to introduce StD, Nintendo Switch, and safety education. During the intervention sessions, facilitators supervised the exercise process, guided participants through the intervention sessions, provided technical guidance on the use of the equipment, ensured participant safety, monitored adherence, and offered general encouragement to maintain participant engagement with the program.

**Figure 1.**
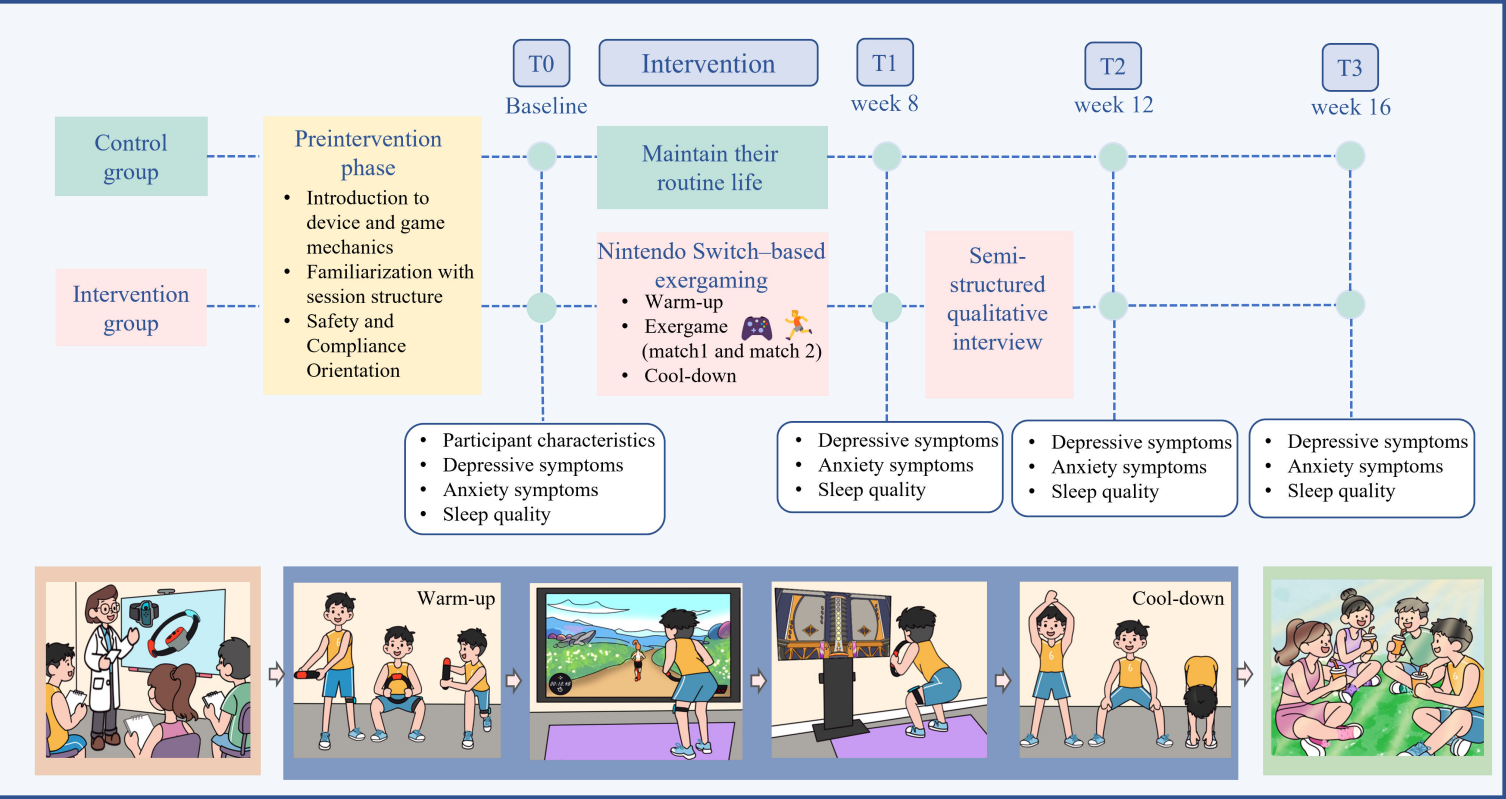
Overview of the Nintendo Switch–based exergaming intervention protocol and assessment schedule in a randomized controlled trial among college students with subthreshold depression.

The intervention program was developed based on existing literature on exergaming, the game category of Ring Fit Adventure (Nintendo), and the exercise prescription guidelines from the American College of Sports Medicine (ACSM), and further refined through group meetings and a pilot study. The total intervention period lasted 16 weeks, consisting of an 8-week intervention followed by an 8-week follow-up period. The intervention was conducted in a psychological and exercise laboratory with sessions held 2‐3 times per week, each lasting 50‐60 minutes. Each session included 3 stages: “Warm-up” (5.5 minutes), “Exergame” (40‐50 minutes) and “Cool-down” (4.5 minutes). The exergaming stage primarily used the adventure mode of Ring Fit Adventure, which integrates aerobic exercise, resistance training, and balance activities through interactive gameplay. This mode incorporates narrative-based progression, in which participants advance through different in-game levels and environments while completing exercise challenges. Participants performed movements using the Ring-Con (Nintendo) and leg strap accessories, enabling movement-based exercises such as squats, running-in-place movements, and upper-body resistance actions that engaged upper-body, core, and lower-limb muscles. During each session, participants progressed through the adventure mode gameplay, which tracks performance across levels and gradually increases exercise intensity through the game’s leveling system and real-time performance feedback. The game provides immediate feedback on movement execution and task completion, allowing participants to adjust their movements and advance to more challenging stages as their performance improved. In addition, 1-2 mini-game challenges were selected each week to enhance motivation and maintain engagement. These mini-games could involve different movement domains, including upper-limb, lower-limb, and balance-related tasks. To further support participant adherence, the exergame content, including adventure mode gameplay and mini-games, was systematically varied across the intervention period, with different gameplay tasks incorporated each week according to the intervention schedule. This structured variation allowed participants to experience diverse exercise challenges while maintaining consistency with the overall training framework. Detailed descriptions of the weekly intervention content and session structure are provided in Figure 1 and Multimedia Appendix 1.

One investigator supervised each session, encouraging participation and recording attendance. Absentees were promptly contacted and encouraged to rejoin. Researchers monitored participants’ well-being and ensured safety. To enhance motivation, weekly results were announced (“Match 1” and “Match 2”), upcoming game content previewed, and small incentives were given.

#### Comparator

Participants in the CG continued with their usual daily activities and were not involved in any additional intervention or structured exercise program during the study period. They did not participate in supervised sessions and received no exercise-related guidance from the research team.

#### Measurements

This study used structured questionnaires to assess the general characteristics of the participants. The primary outcome was depressive symptoms, and the secondary outcomes included anxiety symptoms, sleep quality, and adherence.

#### Depressive Symptoms

Depressive symptoms were screened using the CES-D, developed by Radloff et al [30] and revised by Zhang et al [31]. Responses were rated on a 4-point Likert scale, ranging from “rarely or none of the time” to “most or all of the time.” The total score ranges from 0 to 60, with higher scores indicating greater depression. The Cronbach α was 0.90 [31].

Depressive symptoms were assessed using the Patient Health Questionnaire-9 (PHQ-9) [32], which contains 9 items. Responses were rated on a 4-point Likert scale, from “not at all” to “nearly every day.” The total score ranges from 0 to 27, with higher scores indicating greater severity. The scale has demonstrated good internal consistency (Cronbach α=0.899) in this study.

#### Anxiety Symptoms

Anxiety severity was assessed using the 7-item Generalized Anxiety Disorder Scale (GAD-7), rated on a 4-point Likert scale. The total score ranges from 0 to 21, with cut-off scores of 5, 10, and 15 indicating mild, moderate, and severe anxiety, respectively. In this study, Cronbach α=0.903.

#### Sleep Quality

Sleep quality was measured using the 19-item Pittsburgh Sleep Quality Index (PSQI) [33], which includes open-ended and 4-point Likert scale items. Higher scores indicate worse sleep quality. The PSQI demonstrated good reliability, with a Cronbach α of 0.845 [34] and a test-retest reliability of 0.994. In this study, Cronbach α=0.744.

#### Adherence and Adverse Events

The intervention was considered successful if participants completed at least 80% of the target practice time [15], calculated as the average of the minimum (800 minutes) and maximum (1440 minutes) durations over the 8-week period, yielding a total of 896 minutes. The facilitators supervised participants during the sessions to prevent injuries, overexertion, and excessive gaming behavior. Gameplay was limited to the scheduled intervention sessions, and facilitators monitored participants through direct observation for signs of fatigue or excessive engagement. Any adverse incidents were documented, including their impact and the actions taken to address them.

#### Randomization

Participants were randomly allocated to the IG or CG in a 1:1 ratio using computer-generated permuted block randomization. The random allocation sequence was generated by an independent research coordinator who was not involved in participant recruitment or intervention delivery. Allocation concealment was ensured using sequentially numbered, sealed, opaque envelopes containing the group assignments. After participants completed the baseline assessment, the envelopes were opened in sequence to assign participants to the study groups.

#### Blinding

Due to the nature of the intervention, blinding of participants and intervention providers was not feasible. However, outcome assessors and data analysts were blinded to group allocation to minimize potential bias.

#### Data Collection

Data collection was conducted at 4 time points, including baseline (T0), postintervention (T1, Week 8), one-month follow-up (T2, Week 12), and 2-month follow-up (T3, Week 16), between June 2, 2023 and August 29, 2023. The outcome assessments were conducted by trained assessors, including one psychologist and one registered nurse with clinical experience, who were not involved in delivering the intervention. Before the study began, all assessors received standardized training on the assessment procedures to ensure consistency and reliability. All assessments were conducted using structured checklists in the same simulation environment to maintain procedural consistency. The data collection followed the survey data collection process model [35] (Multimedia Appendix 2). To promote participant retention and minimize loss to follow-up, several strategies were implemented, including supervised intervention sessions, regular reminders before scheduled assessments, flexible scheduling to accommodate participants’ availability, and incentives for completing follow-up assessments.

#### Statistical Analysis

Quantitative data were entered into EpiData (The EpiData Association) and analyzed using SPSS (version 26.0; IBM). Analyses followed the intention-to-treat (ITT) principle. Missing data were assessed using Little’s Missing Completely at Random (MCAR) test. Since the test indicated that the missing data were completely at random, missing values were imputed using the last observation carried forward method. Baseline differences between the 2 groups were assessed using independent 2-tailed *t* tests, Mann-Whitney *U* tests, chi-square tests, or Fisher exact tests, as appropriate. Changes in primary and secondary outcomes from T0 to T1, T2, and T3 were analyzed using generalized estimating equations (GEE) [36] with an exchangeable correlation structure. A *P* value ≤.05 was considered statistically significant.

and analyzed using SPSS (version 26.0; IBM). Analyses followed the ITT principle. Missing data were assessed using Little’s MCAR test. Since the test indicated that the missing data were completely at random, missing values were imputed using the last observation carried forward method. Baseline differences between the 2 groups were assessed using independent *t* tests, Mann-Whitney *U* tests, chi-square tests, or Fisher exact tests, as appropriate. Changes in primary and secondary outcomes from T0 to T1, T2, and T3 were analyzed using GEE [36] with an exchangeable correlation structure. A *P* value ≤.05 was considered statistically significant.

### Qualitative Phase: Semistructured Interviews

#### Research Design

A qualitative descriptive design using qualitative content analysis was adopted to explore participants’ experiences of the Nintendo Switch–based exergaming intervention.

#### Researcher Description

To minimize potential bias, 2 trained researchers (KH and LS) conducted the interviews. The corresponding author (LC), who has extensive experience in qualitative research, facilitated the interviews, while the second author took detailed field notes. Both researchers (KH and LS) were trained to remain neutral and not influence participants’ responses. The research team was balanced in gender (1 female, 1 male) to enhance participant comfort during the interviews. No prior relationship existed between the researchers and participants before the study.

#### Participant Recruitment and Selection

Purposeful sampling was used. An email invitation was sent to 42 adults with StD who had completed the 8-week intervention, encouraging them to share their experiences and reflections. Eligibility criteria for participation in the qualitative study were (1) completion of the 8-week Nintendo Switch–based exercise intervention and (2) provision of informed consent and voluntary agreement to participate. Seventeen participants (2 males and 15 females) from the intervention group (IG) volunteered for semistructured interviews.

#### Data Collection

Semistructured interviews were conducted in a quiet conference room to ensure privacy. Interviews lasted between 20 and 40 minutes. A semistructured interview guide (Multimedia Appendix 3) was used to ensure consistency across interviews while allowing flexibility for participants to elaborate on their experiences. With participants’ consent, all sessions were audio-recorded and transcribed verbatim by a researcher. Participants were assured of anonymity and informed of their right to withdraw at any time. All interviews were transcribed verbatim within 24 hours of completion, and the transcripts were imported into NVivo (12.0; QSR International Pty Ltd), which was used for data management and coding. A second researcher verified all transcripts against the audio recordings to ensure accuracy.

#### Data Analysis

Qualitative data analysis was conducted concurrently with data collection in an iterative manner, allowing emerging insights to inform subsequent interviews and refine thematic exploration until thematic saturation was achieved, that is, no new categories or meaningful information emerged. Qualitative content analysis was used following established methodological guidelines [37,38]. Two researchers independently conducted 2 rounds of open coding, identifying meaningful units of text, and assigning initial codes. These codes were iteratively compared and refined, then grouped into subcategories and further abstracted into generic and main categories through constant comparison. Representative quotations were selected to illustrate and substantiate the identified themes, ensuring interpretations remained grounded in participants’ narratives. Coding results from the 2 researchers were compared throughout the analytic process. Disagreements were documented and resolved through discussion. When consensus could not be reached, a third senior researcher adjudicated the decision to enhance analytical rigor.

#### Methodological Integrity

Methodological integrity was ensured through attention to data adequacy, analytic consistency, and grounding of findings in the data. First, purposive sampling and continued data collection until thematic saturation supported the adequacy of the data. Second, analytic consistency was enhanced through independent coding by 2 researchers, iterative comparison of codes, and resolution of discrepancies through discussion, with adjudication by a third senior researcher when necessary. Third, findings were grounded in participants’ narratives through the use of representative quotations. Finally, field notes, neutral interviewing, and iterative comparison throughout data collection and analysis were used to minimize potential researcher bias and enhance the credibility of the findings.

### Ethical Considerations

Ethical approval was obtained from the Clinical Research Ethics Committee of the School of Nursing, Jilin University (Ref: 2022091401). The study protocol has been published previously [24], providing detailed information on the study design, target population, intervention procedures, and planned data analyses. All study procedures complied with the principles of the Declaration of Helsinki and relevant ethical guidelines for research involving human participants. Written informed consent was obtained from all participants prior to enrollment. To protect participant privacy, all data were deidentified immediately after collection and stored using anonymous identification codes. All analyses were conducted using these coded identifiers, and no personally identifiable information is included in this manuscript or any supplementary materials. Participants who completed the full study protocol, including assessments at T0, T1, T2, and T3, received small gifts after each assessment as appreciation for their participation. Participants who completed all assessments and intervention sessions received an additional gift package valued at approximately Renminbi (RMB) 200, equivalent to approximately US $30.

## Results

### Participant Flow and Recruitment

**Figure 2.**
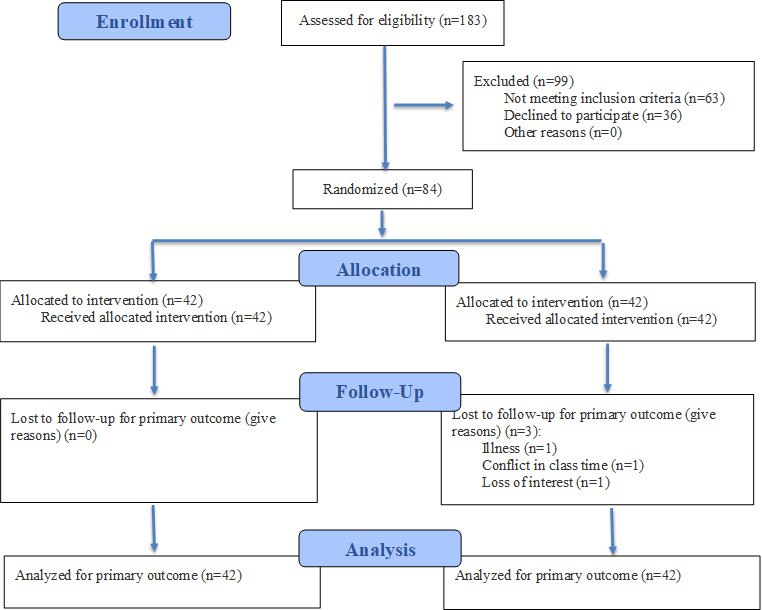
CONSORT (Consolidated Standards of Reporting Trials) 2025 flow diagram of participant recruitment, allocation, follow-up, and analysis.

Figure 2 shows the recruitment and assessment process. Between January and February 2023, eligibility assessments were conducted on 183 potential participants. Of these, 99 individuals were excluded, including 63 who did not meet the inclusion criteria and 36 who declined to participate. A total of 84 eligible participants were randomly assigned in a 1:1 ratio to either the IG (n=42) or the control group (CG; n=42). During the follow-up period, 3/42 (7.1%) participants in the CG discontinued participation at T1 due to illness (n=1), scheduling conflicts with class time (n=1), and loss of interest (n=1). No participants in the IG were lost to follow-up. The overall attrition rate was 3/84 (3.6%). To examine the mechanism of missing data, Little’s MCAR test was conducted for variables included in the outcome analyses (PHQ-9, GAD-7, and PSQI scores), which indicated that the missing data were completely at random (*χ*²_6_=3.606;* P*=.73). Therefore, missing values were handled using the last observation carried forward method under the ITT principle, and all randomized participants (n=84) were included in the final analysis.

### Intervention Delivery and Adherence

The intervention was delivered as planned throughout the 8-week intervention period. A total of 42 participants in the IG received the 8-week Nintendo Switch–based exergaming intervention under the supervision of trained facilitators in the psychological and exercise laboratory setting. All sessions were conducted according to the predefined intervention protocol. Facilitators supervised the exercise sessions, provided technical guidance on the use of the Ring-Con and leg-strap accessories, ensured participant safety, and offered encouragement to support engagement during gameplay. Session attendance and participation were recorded to monitor adherence. Overall, adherence of adults with StD was relatively good; most students (35/42, 83.3%) exceeded 896 minutes of participation time. No adverse event was reported.

### Baseline Data

Table 1 summarizes the baseline characteristics of the participants. The mean age was 23.07 years (SD 1.45), and 83.3% (70/84) of the participants were female. There was no statistically significant difference between the 2 groups in demographic information and outcome variables (*P*>.05).

**Table 1.** Baseline characteristics of participants in the intervention and control groups.

Characteristic	Participants			Test statistics	*P* value
	All (n=84)	IG^a^ (n=42)	CG^b^ (n=42)		
Age (years), mean (SD)	23.07 (1.45)	23.10 (1.48)	23.05 (1.45)	−0.149^c^ (82)	.88
Gender (female), n (%)	70 (83.3)	38 (90.5)	32 (76.2)	3.086^d^ (1)	.08
BMI^e^ (kg/m²), mean (SD)	22.04 (4.2)	22.39 (3.82)	21.70 (4.6)	.233^c^ (82)	.63
Underweight (BMI<18.5), n (%)	12 (14.3)	4 (9.5)	8 (19.1)		
Normal weight (18.5≤ BMI<24.9), n (%)	56 (66.7)	30 (71.4)	26 (61.9)		
Overweight (25≤ BMI<29.9), n (%)	10 (11.9)	5 (11.9)	5 (11.9)		
Obesity (BMI≥30), n (%)	6 (7.1)	3/42 (7.2)	3/42 (7.1)		
Grade, n (%)				11.500^d^ (6)	.07
Undergraduate	58 (69.1)	25 (59.5)	33 (78.6)		
Master degree or higher	26 (31.9)	17 (40.5)	9 (21.4)		
CES-D^f^ score, mean (SD)	20.98 (4.58)	21.57 (4.1)	20.38 (5)	1.193^g^ (82)	.24
PHQ-9^h^ score, median (IQR)	11 (8‐13)	10 (6.75‐13)	11 (9‐13)	.643^i^	.52
GAD-7^j^ score, median (IQR)	6 (4-9)	5.5 (4‐9.25)	6 (4‐8.25)	−.333^i^	.74
PSQI^k^ score, median (IQR)	5.5 (4-7)	6 (5-7)	5 (4-6)	−1.754^i^	.08

a IG: intervention group.

b CG: control group.

c For continuous variables, an independent *t* test was used when variables were compared between the 2 groups.

d For categorical variables, a chi-square test was used when variables were compared between the 2 groups.

e Calculated as weight in kilograms divided by height in meters squared.

f CES-D: Center for Epidemiologic Studies Depression Scale.

g If the scale scores followed a normal distribution, an independent sample *t* test was used.

h PHQ-9: Patient Health Questionnaire-9.

i If the scale scores did not follow a normal distribution, a nonparametric rank sum test was used for statistical analysis.

j GAD-7: Generalized Anxiety Disorder-7.

k PSQI: Pittsburgh Sleep Quality Index.

### Effects on the Depressive Symptoms

**Figure 3.**
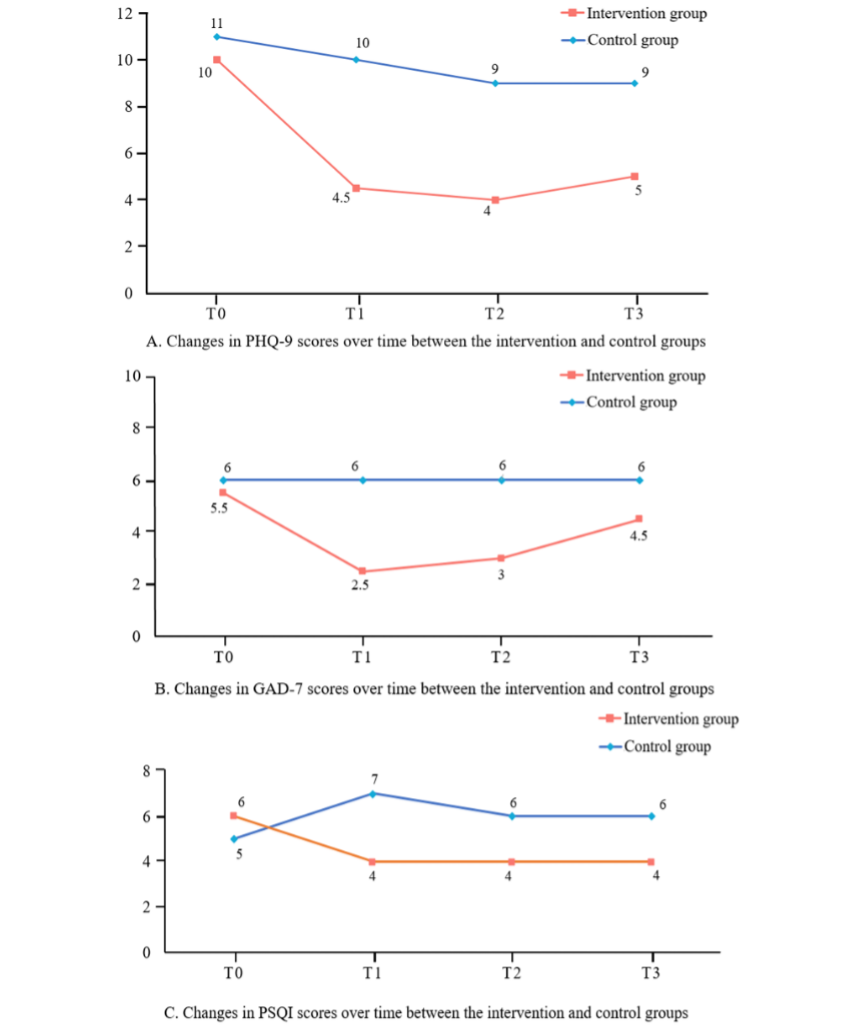
Changes in median scores of depressive symptoms, anxiety symptoms, and sleep quality over time in a randomized controlled trial evaluating the effects of a Nintendo Switch–based exergaming intervention among college students with subthreshold depression. GAD-7: Generalized Anxiety Disorder Scale; PHQ-9: Patient Health Questionnaire-9; PSQI: Pittsburgh Sleep Quality Index.

As shown in Table 2, the statistically significant effects of the group×time interaction between the 2 groups were found in PHQ-9 scores (T1: *β*=−4.07, 95% CI −5.84 to −2.30; *P*<.001; T2: *β*=−4.29, 95% CI −6.14 to −2.43, *P*<.001; T3: *β*=−3.81, 95% CI −5.54 to −2.08; *P*<.001). The results of the pairwise contrast tests are shown in Table 3. The IG showed greater significant improvements in PHQ-9 scores at all T1 (mean difference [MD]=−5.48, 95% CI −6.72 to −4.23; *P*<.001), T2 (MD=−6.10, 95% CI −7.41 to −4.78; *P*<.001), and T3 (MD=−5.79, 95% CI −7.10 to −0.47; *P*<.001) compared with the CG. Figure 3A illustrates the temporal trends of depression outcomes, derived from the results of the GEE analyses.

**Table 2.** Generalized estimating equation (GEE) analysis of changes in depressive symptoms, anxiety symptoms, and sleep quality over time between the intervention and control groups in a randomized controlled trial of a Nintendo Switch–based exergaming intervention among college students with subthreshold depression.

Outcome measure and time	Intervention group (n=42)	Control group (n=42)	Group effect^a^				Time effect^b^				Group×time effect^d^			
	Mean (SD)/ median (IQR)	Mean (SD)/ median (IQR)	*β* (95% CI)	*P* value	Wald *χ*^2^ (df)	*P* value	*β* (95% CI)^c^	*P* value	Wald *χ*^2^ (df)	*P* value	*β* (95% CI)	*P* value	Wald *χ*^2^ *(df)*	*P* value
PHQ-9^e^					29.302 (1)	<.001			101.662 (3)	<.001			28.182 (3)	<.001
T0^f^	10 (6.75‐13)	11 (9-13)	–.48 (–1.92 to 0.96)	.52										
T1^g^	4.5 (1‐8.25)	10 (7-13)					–1.04 (–2.66 to –0.15)	.03			–4.07 (–5.84 to –2.30)	<.001		
T2^h^	4 (1-8)	9 (5.75‐12.5)					–1.81 (–3.13 to –0.49)	.007			–4.29 (–6.14 to –2.43),	<.001		
T3^i^	5 (1-8)	9 (7-11)					–1.98 (–3.12 to –0.85)	.001			–3.81 (–5.54 to –2.08)	<.001		
GAD-7^j^					3.347 (1)	.07			19.684 (3)	<.001			6.734 (3)	.08
T0	5.5 (4‐9.25)	6 (4‐8.25)	.50 (–1.23 to 2.23)	.57										
T1	2.5 (0‐6)	6 (2-8)					–.60 (–2.20 to 1.01)	.47			–2.60 (–4.70 to –0.50)	.02		
T2	3 (0‐7)	6 (0‐7.25)					–1.17 (–2.77 to 0.43)	.15			–2.02 (–4.11 to 0.07),	.06		
T3	4.5 (0‐7)	6 (2.75‐8)					.143 (−1.53 to 1.82)	.87			–2.38 (–4.62 to –0.14)	.04		
PSQI^k^					5.618 (1)	.02			9.824 (3)	.02			23.207 (3)	<.001
T0	6 (5-7)	5 (4-6)	.71 (–0.21 to 1.64)	.131										
T1	4 (2-7)	7 (3-9)					1.31 (0.09 to 2.53)	.04			–2.98 (–4.55 to –1.40)	<.001		
T2	4 (3-5)	6 (3-7)					.19 (−–0.96 to 1.34)	.75			–2.19 (–3.58 to –0.80)	.002		
T3	4 (3-5)	6 (4-7)					.41 (–0.72 to 1.53)	.48			–2.45 (–3.81 to –1.09)	<.001		

a Group effect was defined as group differences at 4 time points (T0, T1, T2, and T3) between the intervention and control groups.

b The baseline measurement (T0) was the reference categories in the generalized estimating equation model and its corresponding null variables.

c Time effect at T1, T2, and T3 was defined as the score variations in the intervention group at these time points compared to T0.

d Group×time effect was defined as the additional score variations in the intervention group compared to the control group.

e PHQ-9: Patient Health Questionnaire-9.

f T0: baseline.

g T1: postintervention (week 8).

h T2: 1-month follow-up (week 12).

i T3: 2-month follow-up (week 16).

j GAD-7: Generalized Anxiety Disorder-7.

k PSQI: Pittsburgh Sleep Quality Index.

**Table 3.** Pairwise comparisons of depressive symptoms, anxiety symptoms, and sleep quality between the intervention and control groups at T1, T2, and T3 in a randomized controlled trial evaluating a Nintendo Switch–based exergaming intervention among college students with subthreshold depression.

Outcomes and comparison		MD^a^ (95% CI)	SE	*P* value
PHQ-9^b^				
T1^c^		–5.48 (–6.72 to –4.23)	.636	<.001
T2^d^		–6.10 (–7.41 to –4.78)	.669	<.001
T3^e^		–5.79 (–7.10 to –0.47)	.670	<.001
GAD-7^f^				
T1		–3.19 (–4.54 to –1.84)	.690	<.001
T2		–3.19 (–4.54 to –1.84)	.687	<.001
T3		–2.24 (–3.73 to –0.75)	.760	.003
PSQI^g^				
T1		–1.67 (–2.66 to –0.67)	.507	.001
T2		–2.00 (–2.78 to –1.22)	.400	<.001
T3		–2.05 (–2.81 to –1.28)	.390	<.001

a MD: mean difference.

b PHQ-9, Patient Health Questionnaire-9.

c T1: postintervention (week 8).

d T2: 1-month follow-up (week 12).

e T3: 2-month follow-up (week 16).

f GAD-7: Generalized Anxiety Disorder-7.

g PSQI: Pittsburgh Sleep Quality Index.

### Effects on the Sleep Quality and Anxiety Symptoms

As shown in Table 2, the group×time interaction had a statistically significant effect on PSQI scores at all follow-up points (T1: *β*=−2.98, 95% CI −4.55 to −1.40; *P*<.001; T2: *β*=−2.19, 95% CI −3.58 to −0.80; *P*=.002; T3: *β*=−2.45, 95% CI −3.81 to −1.09). Anxiety symptoms also improved significantly at T1 (*β*=−2.60, 95% CI −4.70 to −0.50; *P*=.02) and T3 (*β*=−2.38, 95% CI −4.62 to −0.14; *P*=.04). For sleep quality in Table 3, the IG showed greater significant improvements in PSQI scores at all T1 (MD=−1.67, 95% CI −2.66 to −0.67; *P*=.001), T2 (MD=−2.00, 95% CI −2.78 to −1.22; *P*<.001), and T3 (MD=−2.05, 95% CI −2.81 to −1.28; *P*<.001) compared with the CG. In terms of anxiety, there was a significant improvement in the IG compared with the CG also at T1 (MD=−3.19, 95% CI −4.54 to −1.84; *P*<.001), T2 (MD=−3.19, 95% CI −4.54 to −1.84; *P*<.001), and T3 (MD=−2.24, 95% CI −3.73 to −0.75; *P*=.003). Figure 3B-3C illustrates the temporal trends in sleep quality and anxiety outcomes.

### Qualitative Findings

Seventeen adults with StD participated in the semistructured interviews, including 2 males and 15 females, aged 22-29 years (mean 23.18, SD 1.78). The average duration of the interviews was 26.7 minutes. Details are shown in Multimedia Appendix 4.

**Figure 4.**
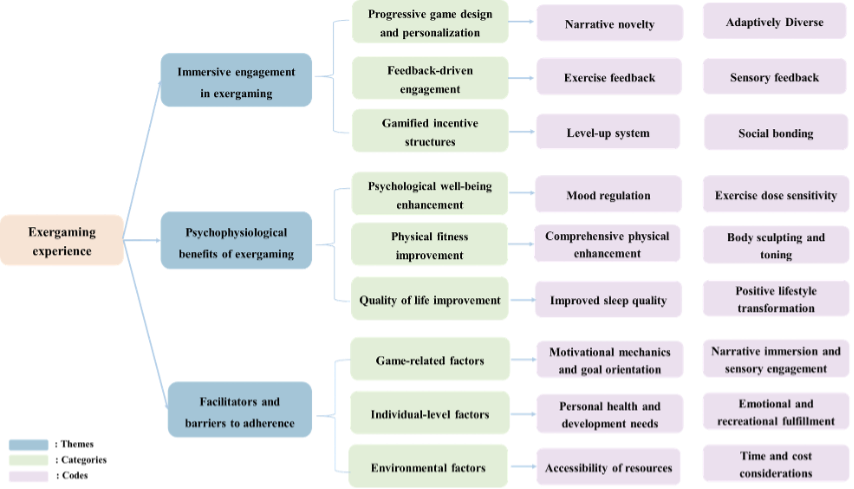
Thematic map displaying themes, categories, and codes.

Three major themes emerged from the semistructured interviews content analysis. The corresponding codes and representative quotations are presented in Table 4. Figure 4 presents the thematic map that shows the relationships among the themes, categories, and codes identified in the qualitative analysis. To protect confidentiality and preserve anonymity, participant quotations are followed by their participant number (*P_n_*).

**Table 4.** Examples of quotations from semistructured interviews (n=17).

Themes, categories, and codes	Examples of quotations
Immersive engagement in exergaming	
Progressive game design and personalization	
Narrative novelty	“The adventure mode unfolds step-by-step with a sense of mystery.” (P2)
Adaptively diverse	“Everyone can customize the skin, hair color, and eyes to match my preferences.” (P4) “The adventure mode lets you choose paths and customize boosts—it’s fun to explore.” (P15)
Feedback-driven engagement	
Sensory feedback	“The stress gage and progress bar helped me track performance and stay motivated.” (P11) “I really liked the sound cues, hearing the music after completing a move gave me a strong sense of satisfaction.” (P13)
Exercise feedback	“Guided movements help ensure accuracy and boost the effectiveness and professionalism of the workout.” (P4)
Gamified incentive structures	
Level-up system	“The level-based challenges, points, and mini-games gave me a real sense of enjoyment and competition.” (P16)
Social bonding	*“*During the workout, the on-screen character moved with me, which gave me a sense of companionship.” (P1)
Psychophysiological benefits of exergaming	
Psychological well-being enhancement	
Mood regulation	“Just want to sweat a bit to release negative emotions.” (P8) “My confidence grew as I completed small goals, I felt I could actually achieve something.” (P9)
Exercise dose sensitivity	“I feel better when the intensity matches my ability. if it’s too high, I lose motivation and can’t stick with it*.*” (P9)
Physical fitness improvement	
Comprehensive physical enhancement	“When climbing stairs or doing physical activities, my heart rate doesn’t rise as quickly — my cardiac function has improved.” (P17)
Body sculpting and toning	“I noticed my body getting firmer, my waist slimmer, which made me feel accomplished and motivated to continue.” (P12)
Quality of life improvement	
Improved sleep quality	“I used to wake up around 1 or 2 AM, but recently I’ve been sleeping through the night.” (P9)
Positive lifestyle transformation	“Since I started training, I feel more energized and motivated. It’s not just exercise, it’s become a fun part of my weekly routine and helps me maintain a healthier lifestyle.” (P14)
Facilitators and barriers to adherence	
Game-related factors	
Motivational mechanics and goal orientation	“Knowing there were rewards really motivated me to participate and give my best.” (P3) “Defeating monsters and earning coins kept me focused and engaged.” (P11)
Narrative immersion and sensory engagement	“The immersive storyline drew me in and kept me engaged.” (P2) “I was always eager to know what would happen next, that curiosity kept me coming back.” (P13)
Individual-level factors	
Personal health and development needs	“I gained weight over the holiday and wanted to lose some fat.” (P4) “I want to improve my skills and prove to my parents I can stick with it—they always say I give up too easily*.*” (P16)
Emotional and recreational fulfillment	“I came here to enrich my after-class life.” (P5) “If I score high and get a small prize, that would make me really happy.” (P6)
Environmental factors	
Accessibility of resources	“It’s hard to exercise in the dorm—too noisy downstairs, too cold outside. Coming here is just right.” (P5) “Running outside means worrying about traffic, people, noise, and safety.” (P8)
Time and cost considerations	“The location and timing work well for me since my classes are nearby, I don’t need to travel far.” (P17) “The staff’s weekly schedule and reminders really helped me stick with it.” (P4)

### Theme 1. Immersive Engagement in Exergaming

#### Progressive Game Design and Personalization

Participants noted that the narrative novelty and diverse and personalized game design fostered curiosity and a strong sense of achievement.

#### Feedback-Driven Engagement

Participants described that sensory feedback and real-time exercise feedback enhanced their movement accuracy and immersion, improving both training effectiveness and safety.

#### Gamified Incentive Structures

Gamified features like points, rankings, and gear upgrades enhanced goal orientation and sustained motivation. Interactive elements such as peer competition and collaboration fostered social connectedness and satisfaction.

### Theme 2. Psychophysiological Benefits of Exergaming

#### Psychological Well-Being Enhancement

Engaging in exergames was perceived to improve psychological well-being by relieving negative emotions, enhancing self-efficacy, and supporting mood regulation through appropriate exercise intensity.

#### Physical Fitness Improvement

Participants reported that exergames enhanced physical fitness by combining aerobic, strength, and flexibility training, improving endurance, muscle tone, and overall body control.

#### Quality of Life Improvement

Participants noted that exergames enhanced their quality of life by promoting energy, mood, metabolism, appetite, and sleep through an engaging blend of exercise and entertainment.

### Theme 3. Facilitators and Barriers to Adherence

#### Game-Related Factors

Core game elements, including goals, challenges, rewards, narrative, social interaction, and audiovisual effects, enhanced immersion, enjoyment, and engagement, thereby fostering player motivation and adherence.

#### Individual-Level Factors

Participants identified health improvement, recreational enjoyment, self-challenge, skill development, and emotional fulfillment as key individual drivers of sustained engagement.

#### Environmental Factors

Participants indicated that accessibility of equipment and venues, along with time availability and financial cost, were key environmental factors influencing adherence to exergames.

## Discussion

### Principal Findings

This study evaluated the effectiveness of a Nintendo Switch–based exergaming program for adults with StD using a mixed methods design. The results showed that participants who engaged in the exergaming program experienced significant improvements in depressive symptoms and sleep quality, along with reductions in anxiety symptoms, compared with the CG. The qualitative findings further complemented the quantitative results by providing deeper insights into participants’ experiences with the exergaming intervention. Participants described immersive engagement during gameplay, perceived psychophysiological benefits, and multiple facilitators and barriers influencing adherence (eg, game-related, individual-level, and environmental factors), which helped contextualize the observed improvements in depressive symptoms, anxiety symptoms, and sleep quality.

### Interpretation

Our findings indicate that the 8-week Nintendo Switch–based exergaming intervention significantly improved depressive symptoms among adults with StD, with improvements maintained at both 4-week and 8-week follow-up assessments. Consistent with previous literature, regular physical activity has been widely recognized as an effective nonpharmacological strategy for improving depressive symptoms [39]. These benefits may be partly explained by biological mechanisms whereby exercise promotes neuroplasticity and neurogenesis through the regulation of neurotrophic factors and modulates the synthesis and release of key neurotransmitters involved in mood regulation [40]. In addition to these physiological mechanisms, exercise may also contribute to mood improvement through behavioral and psychological pathways, including behavioral activation, improved sleep, and enhanced psychological well-being [40]. However, traditional exercise programs often face challenges related to low motivation and poor long-term adherence, particularly among individuals experiencing depressive symptoms [41].

Exergames, as a specific type of serious game that integrates physical activity with interactive gameplay, may help address these challenges by enhancing engagement and motivation [8]. From the perspective of behavioral activation theory [42], individuals with StD often experience reduced activity levels and diminished exposure to rewarding experiences [43]. Exergames such as Ring Fit Adventure provide structured, goal-oriented tasks, real-time feedback, and reward systems that encourage participants to re-engage in meaningful and rewarding activities [44]. Through repeated participation, these mechanisms may help break cycles of avoidance and inactivity and promote behavioral activation [45]. In addition, several design features of exergames may further contribute to their psychological benefits [46]. As reflected in participants’ postintervention interviews, game elements such as progressive game design and personalization, feedback-driven engagement, and gamified incentive structures may enhance users’ self-efficacy and perceived competence. According to social cognitive theory, higher self-efficacy is an important determinant of sustained behavioral engagement [47]. At the same time, gamified reward structures and immersive gameplay may increase enjoyment and intrinsic motivation, thereby promoting sustained participation in physical activity [48]. This mechanism was further reflected in participants’ feedback; for example, one participant noted that “the level-based challenges, points, and mini-games gave me a real sense of enjoyment and competition,” highlighting how game-based elements can transform exercise into a more engaging and motivating experience.

The present findings differ from those reported by Wu et al [16], who reported less favorable mood-related outcomes following a 4-week Ring Fit Adventure intervention in university students. One possible explanation is intervention duration. Longer intervention periods may provide more time for participants to establish regular engagement and benefit from repeated behavioral reinforcement [49], thereby supporting more stable behavioral and emotional change, consistent with behavior change theory [50]. This interpretation is also consistent with our previous meta-analysis, which suggested that longer-duration exergame interventions were associated with greater improvements in depressive symptoms [28]. Another possible explanation relates to differences in sample characteristics. The present study included a higher proportion of female participants. Previous research has suggested that women may demonstrate higher adherence to structured health programs, which could influence intervention outcomes [51]. Therefore, the gender composition of the sample may have partly contributed to the more favorable results observed in the present study.

A slight rebound in depressive symptoms was observed at the T2 and T3 follow-up stage. One possible explanation is that the beneficial effects of the intervention gradually attenuated after the withdrawal of structured guidance and routine participation. In addition, the later follow-up period overlapped with examination periods for many students, which may have increased academic stress and negatively affected mental health [52].

Although the group×time result for GAD-7 was not statistically significant, pairwise comparisons revealed significantly greater anxiety reductions in the IG at all time points. This suggests that Nintendo Switch–based exergaming may still exert beneficial effects. The finding is broadly consistent with a prior study [53]. One possible explanation lies in the differential sensitivity of anxiety to behavioral activation. While both anxiety and depression involve disrupted emotional regulation, anxiety is often more reactive to acute stressors and environmental uncertainty [54]. Exergames may contribute to anxiety reduction by providing structured physical routines, immersive attentional distraction, and rhythmic motor engagement [55].

The study also suggested that exergaming intervention showed potential to improve sleep quality, although a previous study using a similar intervention did not report statistically significant effects [16]. These improvements may reflect both psychological and physiological pathways. Psychologically, reductions in depressive symptoms, anxiety, and stress may indirectly contribute to better sleep [56,57]. Physiologically, exergaming may support sleep through mechanisms related to thermoregulation, autonomic function, and overall physical activation [58,59]. In addition, qualitative findings suggested that participants experienced relaxation and enhanced well-being during the intervention, which may also have contributed to improved sleep.

The study demonstrated high adherence and no adverse events, indicating the intervention’s safety and feasibility. This strong engagement was likely supported by both the design features of the exergame and the structured implementation context [46]. On the one hand, game elements such as interactive feedback, clear goals, progressive challenges, and an engaging narrative may have enhanced participants’ motivation and enjoyment [44]. On the other hand, the intervention was delivered in a structured laboratory environment with scheduled sessions, facilitator supervision, and regular support from the research team, which likely further promoted adherence and sustained participation [60]. Accordingly, the observed effects may reflect the combined influence of both game design and human support.

### Limitations and Future Directions

This study has several limitations. First, the sample consisted exclusively of university students, whose stress patterns, sleep routines, and lifestyle characteristics may differ from those of the broader adult population, particularly during examination periods, thus limiting the generalizability of the findings. Second, the intervention was delivered in a structured laboratory environment with scheduled sessions and facilitator supervision. Although this likely supported adherence and intervention fidelity, it differs from the typical home-based use of Ring Fit Adventure. Therefore, caution is needed when generalizing the findings to less controlled real-world settings. Third, the IG and CG differed in the degree of structure and contact with the research team. Participants in the IG received supervised sessions and regular support, whereas the CG continued usual activities without comparable contact. As such, some of the observed effects may have been influenced not only by the exergaming intervention itself but also by differences in attention and support. Fourth, because the intervention combined both game-based design features and facilitator support, it was not possible to fully disentangle the relative contributions of human scaffolding and game design to the observed outcomes. This distinction is important for understanding the scalability of the intervention in less supported settings. Fifth, the follow-up period was limited to 8 weeks. As such, the long-term sustainability of the observed improvements in depressive symptoms and sleep quality remains uncertain. Future studies with extended follow-up periods are warranted to determine whether these benefits persist over time. Sixth, due to the nature of the intervention, blinding of participants and intervention providers was not feasible. This may have introduced potential performance or expectancy biases, as participants aware of receiving the intervention might have reported greater improvements [61]. However, outcome assessors and data analysts were blinded to group allocation to minimize potential bias. Future studies could further strengthen methodological rigor by incorporating additional objective outcome measures.

### Innovation and Contribution

This study makes several contributions to the exergame literature. First, it extends the evidence by focusing specifically on adults with StD, a population at elevated risk of developing MDD but underrepresented in exergaming research. In contrast to many previous studies that have primarily targeted older adults or specific clinical populations, the present study examined the potential of exergaming as an early preventive intervention for adults with StD. Second, this study was designed as a mixed methods exergame effectiveness trial, combining longitudinal outcome evaluation with qualitative inquiry. This approach not only assessed whether the intervention was beneficial but also provided insight into how participants experienced the program and which factors influenced engagement and adherence. Third, the findings contribute to the broader exergames for mental health field by showing how design features such as progressive challenges, feedback, and gamified incentives may support behavioral activation, self-efficacy, and sustained participation. Finally, from a practical perspective, the study suggests that commercially available exergaming platforms such as Nintendo Switch–based exergaming intervention offer a feasible, engaging, and potentially scalable digital strategy for supporting mental health and sleep outcomes in adults with StD.

### Conclusion

This study identified the positive effects of an 8-week Nintendo Switch–based exergaming intervention in improving depressive symptoms, anxiety, and sleep quality among adults with StD. Qualitative findings further revealed that immersive engagement, perceived psychophysiological benefits, and multifaceted adherence facilitators ranging from gameplay design to individual and environmental factors played a key role in sustaining participation and enhancing intervention effectiveness. Together, these findings suggest that gamified physical activity may represent a feasible and engaging approach for promoting mental well-being in adults with StD. By integrating interactive gameplay with exercise, commercially available exergaming platforms may offer a promising and accessible strategy for early mental health support in real-world settings.

## Supplementary material

10.2196/80937 Multimedia Appendix 1 Intervention manual of the Nintendo Switch–based exergaming program.

10.2196/80937 Multimedia Appendix 2 Survey data collection process model.

10.2196/80937 Multimedia Appendix 3 Interview guide of the qualitative phase.

10.2196/80937 Multimedia Appendix 4 Sociodemographic characteristics of the participants (n = 17).

10.2196/80937 Checklist 1 CONSORT checklist.

10.2196/80937 Checklist 2 CONSORT-eHEALTH checklist (V 1.6.1).

10.2196/80937 Checklist 3 JARS-Qual checklist.

## Abbreviations

ACSM: American College of Sports Medicine
CES-D: Center for Epidemiologic Studies Depression Scale
CG: control group
CONSORT: Consolidated Standards of Reporting Trials
CONSORT-EHEALTH: Consolidated Standards of Reporting Trials of Electronic and Mobile Health Applications and Online Telehealth
*DSM-5*: *Diagnostic and Statistical Manual of Mental Disorders, Fifth Edition*
GAD-7: Generalized Anxiety Disorder-7
GEE: generalized estimating equation
HAMD-24: 24-item Hamilton Depression Rating Scale
IG: intervention group
ITT: intention-to-treat
JARS-Qual: Journal Article Reporting Standards for Qualitative Research
MCAR: Missing Completely at Random
MD: mean difference
MDD: major depressive disorder
PHQ-9: Patient Health Questionnaire-9
PSQI: Pittsburgh Sleep Quality Index
RCT: randomized controlled trial
StD: subthreshold depression
