# Supplementary material for: Nintendo Switch–Based Exergaming for Subthreshold Depression: Mixed Methods Randomized Controlled Trial
Source: JMIR Serious Games. 2026 Jun 5;14:e80937. doi: 10.2196/80937 (PMC13240639; doi:10.2196/80937)
Supplement: Multimedia Appendix 1 [file games-v14-e80937-s001.docx]

# Multimedia Appendix 1.

Intervention Manual of the Nintendo Switch™-Based Exergaming Program

| **Session 1-2**  *Introduction to Subthreshold Depression and Nintendo Switch™* |
| --- |
| ***Aims***   - Introduce the Subthreshold Depression, and discuss the prognosis, symptom manifestations, subsequent impacts, risk factors, and treatment options of Subthreshold Depression. - Invite the participants to share their Subthreshold Depression experience and the subsequent impacts on everyday living. - Enhance participants’ confidence and competence in using the Nintendo Switch™ system and exergaming equipment through structured demonstrations and hands-on practice. - Equip participants with basic skills in self-monitoring physical responses and recognizing potential safety risks during exergaming. |
| ***Content***   1. **Ice breaking (10 minutes)**  - Self-introduction - Brief orientation about the study aims and the themes of each session  1. **Introduction to** **Subthreshold Depression (30 minutes)**  - What is Subthreshold Depression? - Discuss the nature, prognosis, and risk factors of Subthreshold Depression - Nature (prevalence, diagnostic criteria, causes) - Prognosis (continuum of depression) - Risk factors (loneliness, social isolation, stressful life events etc.) - Reflection and experience sharing - Discuss the impacts of cognitive/ neuropsychiatric symptoms on daily living - Low energy or fatigue → reduced task efficiency and physical disengagement - Loss of interest in enjoyable activities → social withdrawal → loneliness - Difficulty concentrating → errors in tasks or studies → self-doubt - Irritability or emotional sensitivity → interpersonal conflict → guilt - Sleep disturbances (insomnia or hypersomnia) → daytime dysfunction → frustration  1. **Introduction to Nintendo Switch™ (50 minutes)**  - **Overview and Introduction** - Briefly introduce the Nintendo Switch™ console, Joy-Con, Ring-Con, Leg Strap, and Ring Fit Adventure game. - Explain the rationale for choosing this exergaming platform in psychological and physical health interventions. - **Live Demonstration** - System startup and game loading - Attaching Joy-Con to Ring-Con and Leg Strap - Basic interface navigation (e.g., home screen, user profile, game menu) - Starting and pausing the game - Performing simple exercises in the game (e.g., squats, knee lifts) - **Hands-On Practice** - **Debrief and Q&A** - Invite participants to share feelings, questions, or challenges encountered.  1. **Safety education (20 minutes)**  - **Introduction & Rationale** - **Physical Safety Guidelines** - Before exercise: Wear proper attire, hydrate, clear exercise space. - During: Maintain posture, avoid overexertion, follow in-game guidance. - After: Cool down, monitor for soreness, dizziness, or fatigue. - **Psychological Safety Reminders** - Remind participants that frustration during the game is normal. - Emphasize self-paced progression and non-competitive engagement. - Encourage communication with facilitators if they feel anxious, embarrassed, or unmotivated. - **Emergency Response and Monitoring Plan** - Introduce on-site support staff and symptom reporting procedure. - Demonstrate how to stop gameplay anytime. - Distribute emergency contact cards.  1. **Tips for maintaining mental health (10 minutes)**  - **Maintain a healthy diet** - **Engage in an active lifestyle** - **Practice emotional expression** - **Improve sleeping quality** - **Limit excessive screen time** |

| **Session 3**  *Week 1 of Intervention* |
| --- |
| ***Aims***   - Initiate physical activity to reduce depressive and anxiety symptoms. - Promote relaxation and physical exertion to improve sleep quality. |
| ***Content***   1. **Warm-up Phase (5-6 minutes)**  - **Dynamic Stretching** - **Arm Stretching (Raise Both Arms and Push Up)** - **Abdominal Stretching (Reach and Raise Arms Sideways)** - **Leg Stretching (Step Forward, Squat)** - **Yoga Stretching (Twist and Measure Angular Pose)**  1. **Exergaming Session (40-50 minutes)**  - **Match 1：Tracks the participant’s performance across levels****(30-40 minutes)** - Adventure Mode - **Match 2:  Involves 1–2 rotating mini-game challenges from Ring Fit Adventure™ each week to sustain motivation and promote active engagement (10 minutes).** - Pectoral Muscle Training - Whac-A-Mole - Parachute - Squat Jump - Ichino's Land  1. **Cool-down Phase (4-5 minutes)**  - **Stretching exercises focus on relaxing the abdominal, lumbar, and leg areas** |

| **Session 4**  *Week 2 of Intervention* |
| --- |
| ***Aims***   - Initiate physical activity to reduce depressive and anxiety symptoms. - Promote relaxation and physical exertion to improve sleep quality. |
| ***Content***   1. **Warm-up Phase (5-6 minutes)**  - **Dynamic Stretching** - **Arm Stretching (Raise Both Arms and Push Up)** - **Abdominal Stretching (Reach and Raise Arms Sideways)** - **Leg Stretching (Step Forward, Squat)** - **Yoga Stretching (Twist and Measure Angular Pose)**  1. **Exergaming Session (40-50 minutes)**  - **Match 1：Tracks the participant’s performance across levels (30-40 minutes)** - Adventure Mode - **Match 2:  Involves 1–2 rotating mini-game challenges from Ring Fit Adventure™ each week to sustain motivation and promote active engagement (10 minutes).** - Latissimus Dorsi Challenge - Deep Squat Bounce - Box Breaking - Balance Walking - Spoda Highway  1. **Cool-down Phase (4-5 minutes)**  - **Stretching exercises focus on relaxing the abdominal, lumbar, and leg areas** |

| **Session 5**  *Week 3 of Intervention* |
| --- |
| ***Aims***   - Initiate physical activity to reduce depressive and anxiety symptoms. - Promote relaxation and physical exertion to improve sleep quality. |
| ***Content***   1. **Warm-up Phase (5-6 minutes)**  - **Dynamic Stretching** - **Arm Stretching (Twisting Arms)** - **Abdominal Stretching (Triceps Stretch, Raise Both Arms Sideways)** - **Leg Stretching (Leg Lifts, Forward Fold Pose)** - **Yoga Stretching (Morning Pose With Raised Arms)**  1. **Exergaming Session (40-50 minutes)**  - **Match 1：Tracks the participant’s performance across levels (30-40 minutes)** - Adventure Mode - **Match 2:  Involves 1–2 rotating mini-game challenges from Ring Fit Adventure™ each week to sustain motivation and promote active engagement (10 minutes).** - Latissimus Dorsi Training - Quadriceps Training - Whac-A-Mole - Parachute - Floating World Hall  1. **Cool-down Phase (4-5 minutes)**  - **Stretching exercises focus on relaxing the abdominal, lumbar, and leg areas** |

| **Session 6**  *Week 4 of Intervention* |
| --- |
| ***Aims***   - Initiate physical activity to reduce depressive and anxiety symptoms. - Promote relaxation and physical exertion to improve sleep quality. |
| ***Content***   1. **Warm-up Phase (5-6 minutes)**  - **Dynamic Stretching** - **Arm Stretching (Twisting Arms)** - **Abdominal Stretching (Triceps Stretch, Raise Both Arms Sideways)** - **Leg Stretching (Leg Lifts, Forward Fold Pose)** - **Yoga Stretching (Morning Pose With Raised Arms)**  1. **Exergaming Session (40-50 minutes)**  - **Match 1：Tracks the participant’s performance across levels (30-40 minutes)** - Adventure Mode - **Match 2:  Involves 1–2 rotating mini-game challenges from Ring Fit Adventure™ each week to sustain motivation and promote active engagement (10 minutes).** - Erector Spinae Training - Squat Jumps - Inner Thigh Rider - Raise Arms High (Get) - Little Bridge Trail  1. **Cool-down Phase (4-5 minutes)**  - **Stretching exercises focus on relaxing the abdominal, lumbar, and leg areas** |

| **Session 7**  *Week 5 of Intervention* |
| --- |
| ***Aims***   - Initiate physical activity to reduce depressive and anxiety symptoms. - Promote relaxation and physical exertion to improve sleep quality. |
| ***Content***   1. **Warm-up Phase (5-6 minutes)**  - **Dynamic Stretching** - **Arm Stretching (Twist Arms, Hero 2 Pose)** - **Abdominal Stretching (Bent-over Row)** - **Leg Stretching (Step Up, Leg Lift)** - **Yoga Stretching (Morning Pose With Raised Arms)**  1. **Exergaming Session (40-50 minutes)**  - **Match 1：Tracks the participant’s performance across levels (30-40 minutes)** - Adventure Mode - **Match 2:  Involves 1–2 rotating mini-game challenges from Ring Fit Adventure™ each week to sustain motivation and promote active engagement (10 minutes).** - Endless Challenge for Deltoids - Hand Pull Embryo - Inner Thigh Rider - Coin Run - Running Bridge  1. **Cool-down Phase (4-5 minutes)**  - **Stretching exercises focus on relaxing the abdominal, lumbar, and leg areas** |

| **Session 8**  *Week 6 of Intervention* |
| --- |
| ***Aims***   - Initiate physical activity to reduce depressive and anxiety symptoms. - Promote relaxation and physical exertion to improve sleep quality. |
| ***Content***   1. **Warm-up Phase (5-6 minutes)**  - **Dynamic Stretching** - **Arm Stretching (Twist Arms, Hero 2 Pose)** - **Abdominal Stretching (Bent-over Row)** - **Leg Stretching (Step Up, Leg Lift)** - **Yoga Stretching (Morning Pose With Raised Arms)**  1. **Exergaming Session (40-50 minutes)**  - **Match 1：Tracks the participant’s performance across levels (30-40 minutes)** - Adventure Mode - **Match 2:  Involves 1–2 rotating mini-game challenges from Ring Fit Adventure™ each week to sustain motivation and promote active engagement (10 minutes).** - Endless Challenge for Latissimus Dorsi - Hand Pull Embryo - Waist Twist Punch - Coin Run - Floating World Hall  1. **Cool-down Phase (4-5 minutes)**  - **Stretching exercises focus on relaxing the abdominal, lumbar, and leg areas** |

| **Session 9**  *Week 7 of Intervention* |
| --- |
| ***Aims***   - Initiate physical activity to reduce depressive and anxiety symptoms. - Promote relaxation and physical exertion to improve sleep quality. |
| ***Content***   1. **Warm-up Phase (5-6 minutes)**  - **Dynamic Stretching** - **Arm Stretching (Shoulder Press, Raise Arms Twist)** - **Abdominal Stretching (Bent-over Row)** - **Leg Stretching (Wide Squat, Chair Pose)** - **Yoga Stretching (Mountain Pose)**  1. **Exergaming Session (40-50 minutes)**  - **Match 1：Tracks the participant’s performance across levels (30-40 minutes)** - Adventure Mode - **Match 2:  Involves 1–2 rotating mini-game challenges from Ring Fit Adventure™ each week to sustain motivation and promote active engagement (10 minutes).** - Quadriceps Challenge - Disk Punch - Coin Run - Hand Pull Embryo - Ignoto Land  1. **Cool-down Phase (4-5 minutes)**  - **Stretching exercises focus on relaxing the abdominal, lumbar, and leg areas** |

| **Session 10**  *Week 8 of Intervention* |
| --- |
| ***Aims***   - Initiate physical activity to reduce depressive and anxiety symptoms. - Promote relaxation and physical exertion to improve sleep quality. |
| ***Content***   1. **Warm-up Phase (5-6 minutes)**  - **Dynamic Stretching** - **Arm Stretching (Shoulder Press, Raise Arms Twist)** - **Abdominal Stretching (Bent-over Row)** - **Leg Stretching (Wide Squat, Chair Pose)** - **Yoga Stretching (Mountain Pose)**  1. **Exergaming Session (40-50 minutes)**  - **Match 1：Tracks the participant’s performance across levels (30-40 minutes)** - Adventure Mode - **Match 2:  Involves 1–2 rotating mini-game challenges from Ring Fit Adventure™ each week to sustain motivation and promote active engagement (10 minutes).** - Chest Press Challenge - Parachute - Box Break - Disk Strike - Spada Highway  1. **Cool-down Phase (4-5 minutes)**  - **Stretching exercises focus on relaxing the abdominal, lumbar, and leg areas** |
