# Supplementary material for: Nintendo Switch–Based Exergaming for Subthreshold Depression: Mixed Methods Randomized Controlled Trial
Source: JMIR Serious Games. 2026 Jun 5;14:e80937. doi: 10.2196/80937 (PMC13240639; doi:10.2196/80937)
Supplement: Multimedia Appendix 2 [file games-v14-e80937-s002.docx]

# Multimedia Appendix 2.

Survey data collection process model

**
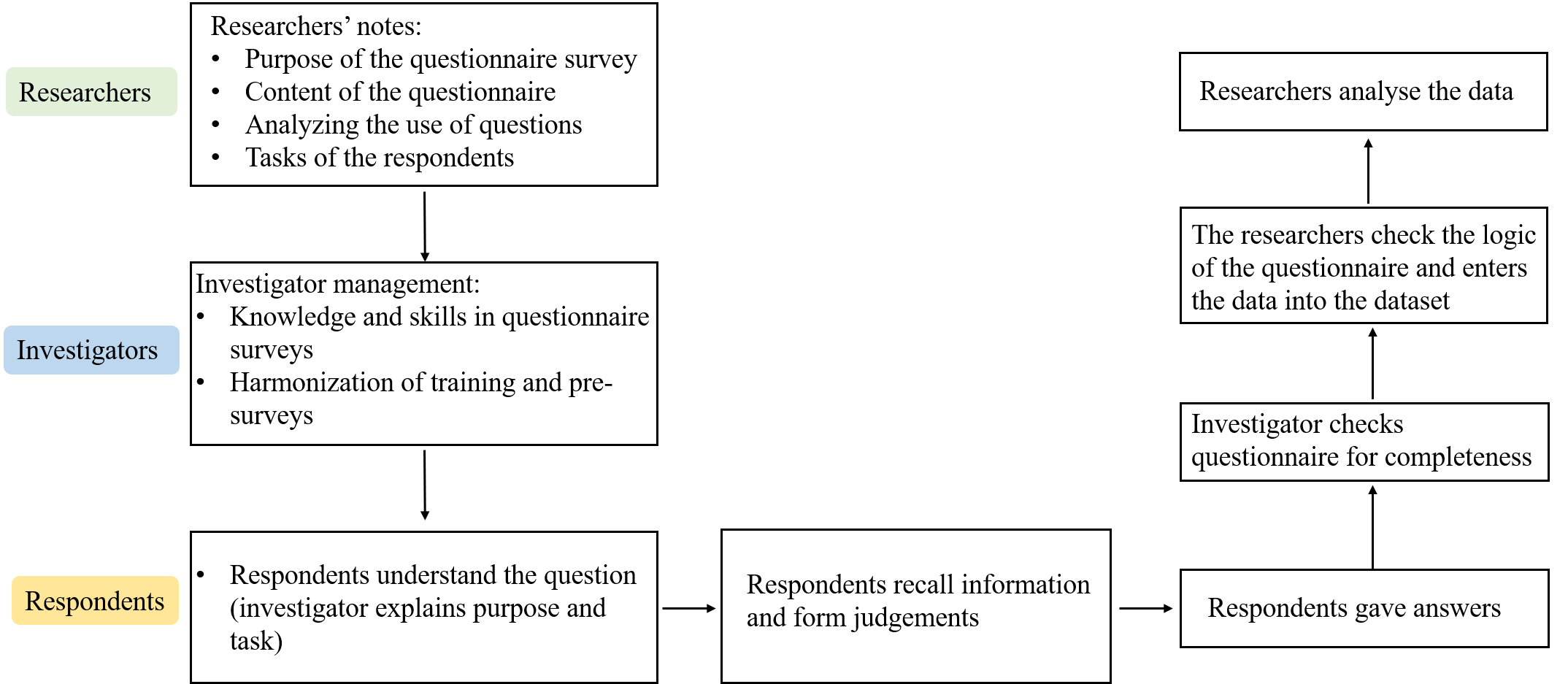
**

**Figure 1.** Survey data collection process model
