# Supplementary material for: Nintendo Switch–Based Exergaming for Subthreshold Depression: Mixed Methods Randomized Controlled Trial
Source: JMIR Serious Games. 2026 Jun 5;14:e80937. doi: 10.2196/80937 (PMC13240639; doi:10.2196/80937)
Supplement: Multimedia Appendix 3 [file games-v14-e80937-s003.docx]

# Multimedia Appendix 3.

Interview guide of the qualitative phase

| **Number** | **Questions** |
| --- | --- |
| 1 | What was your initial reason (or motivation) for participating in the exergame intervention? Please describe your reasons in order of importance. |
| 2 | What are your feelings after attending the exergame intervention? |
| 3 | What challenges have you encountered when participating in the exergame intervention? What was the biggest challenge? How was it resolved? Please provide examples and details. |
| 4 | Would you like to participate in other activities similar to the exergame intervention in the future? Would you recommend the exergame intervention to other people? Please state specific reasons. |
| 5 | What traditional exercise activities have you participated in before? In what ways do you think this combination of exercise and play could benefit you more than traditional exercise methods? |
| 6 | What kind of support and assistance in the exergame intervention would you like to receive in the future? Please be specific about what you think needs to be improved and refined. |
| 7 | Is there anything else you would like to add to today's conversation? |
