# Supplementary material for: Nintendo Switch–Based Exergaming for Subthreshold Depression: Mixed Methods Randomized Controlled Trial
Source: JMIR Serious Games. 2026 Jun 5;14:e80937. doi: 10.2196/80937 (PMC13240639; doi:10.2196/80937)
Supplement: Multimedia Appendix 4 [file games-v14-e80937-s004.docx]

# Multimedia Appendix 4.

Socio-demographic characteristics of the participants (n = 17)

| **Number** | **Sex** | **Age (yrs)** | **Grade** | **BMI** | **History of depression** |
| --- | --- | --- | --- | --- | --- |
| P1 | M | 29 | Doctor | 21.22 | None |
| P2 | F | 24 | Master | 20.20 | None |
| P3 | F | 23 | Master | 24.77 | None |
| P4 | F | 22 | Undergraduate | 21.26 | None |
| P5 | F | 22 | Undergraduate | 21.63 | None |
| P6 | F | 22 | Undergraduate | 18.83 | None |
| P7 | F | 25 | Undergraduate | 21.38 | None |
| P8 | F | 24 | Master | 19.95 | None |
| P9 | M | 23 | Master | 20.80 | None |
| P10 | F | 22 | Undergraduate | 27.99 | None |
| P11 | F | 24 | Master | 26.04 | None |
| P12 | F | 22 | Undergraduate | 19.90 | None |
| P13 | F | 23 | Undergraduate | 20.55 | None |
| P14 | F | 22 | Undergraduate | 17.76 | None |
| P15 | F | 23 | Master | 21.14 | None |
| P16 | F | 22 | Undergraduate | 16.33 | None |
| P17 | F | 22 | Undergraduate | 19.81 | None |

Note.

Abbr. yrs, years old; BMI, Body Mass Index; M, Male; F, Female.
