# Supplementary material for: Nintendo Switch–Based Exergaming for Subthreshold Depression: Mixed Methods Randomized Controlled Trial
Source: JMIR Serious Games. 2026 Jun 5;14:e80937. doi: 10.2196/80937 (PMC13240639; doi:10.2196/80937)
Supplement: Checklist 3 [file games-v14-e80937-s007.pdf]

JARS–**Qual** | Table 1

Information Recommended for Inclusion in Manuscripts That Report Primary Qualitative Research

Title Page

Title

- Identify key issues/topic under consideration.

Author Note

- Acknowledge funding sources or contributors.
- Acknowledge conflicts of interest, if any.

Abstract

- State the problem/question/objectives under investigation.
- Indicate the study design, including types of participants or data sources, analytic strategy, main results/findings, and main implications/significance.
- Identify five keywords.

Guidance for Authors

- Consider including at least one keyword that describes the method and one that describes the types of participants or phenomena under investigation.
- Consider describing your approach to inquiry when it will facilitate the review process and intelligibility of your paper. If your work is not grounded in a specific approach to inquiry or your approach would be too complicated to explain in the allotted word count, however, it would not be advisable to provide explication on this point in the abstract.

Introduction

Description of Research Problem or Question

- Frame the problem or question and its context.
- Review, critique, and synthesize the applicable literature to identify key issues/debates/theoretical frameworks in the relevant literature to clarify barriers, knowledge gaps, or practical needs.

Guidance for Reviewers

- The introduction may include case examples, personal narratives, vignettes, or other illustrative material.

Study Objectives/Aims/Research Goals

- State the purpose(s)/goal(s)/aim(s) of the study.
- State the target audience, if specific.
- Provide the rationale for fit of design used to investigate this purpose/goal (e.g., theory building, explanatory, developing understanding, social action, description, highlighting social practices).

Study Objectives/Aims/Research Goals *(continued)*

- Describe the approach to inquiry, if it illuminates the objectives and research rationale (e.g., descriptive, interpretive, feminist, psychoanalytic, postpositivist, critical, postmodern, constructivist, or pragmatic approaches).

Guidance for Authors

- If relevant to objectives, explain the relation of the current analysis to prior articles/publications.

Guidance for Reviewers

- Qualitative studies often legitimately need to be divided into multiple manuscripts because of journal article page limitations, but each manuscript should have a separate focus.
- Qualitative studies tend not to identify hypotheses, but rather research questions and goals.

Method

Research Design Overview

- Summarize the research design, including data-collection strategies, data-analytic strategies, and, if illuminating, approaches to inquiry (e.g., descriptive, interpretive, feminist, psychoanalytic, postpositivist, critical, postmodern, constructivist, or pragmatic approaches).
- Provide the rationale for the design selected.

Guidance for Reviewers

- Method sections can be written in a chronological or narrative format.
- Although authors provide a method description that other investigators should be able to follow, it is not required that other investigators arrive at the same conclusions but rather that the method description leads other investigators to conclusions with a similar degree of methodological integrity.
- At times, elements may be relevant to multiple sections and authors need to organize what belongs in each subsection in order to describe the method coherently and reduce redundancy. For instance, the overview and the objectives statement may be presented in one section.
- Processes of qualitative research are often iterative versus linear, may evolve through the inquiry process, and may move between data collection and analysis in multiple formats. As a result, data collection and analysis sections might be combined.
- For the reasons above and because qualitative methods often are adapted and combined creatively, requiring detailed description and rationale, an average qualitative Method section typically is longer than an average quantitative Method section.

© 2024 American Psychological Association

Study Participants or Data Sources

Researcher Description

- Describe the researchers' backgrounds in approaching the study, emphasizing their prior understandings of the phenomena under study (e.g., interviewers, analysts, or research team).
- Describe how prior understandings of the phenomena under study were managed and/or influenced the research (e.g., enhancing, limiting, or structuring data collection and analysis).

Guidance for Authors

- Prior understandings relevant to the analysis could include, but are not limited to, descriptions of researchers' demographic/cultural characteristics, credentials, experience with phenomena, training, values, and/or decisions in selecting archives or material to analyze.

Guidance for Reviewers

- Researchers differ in the extensiveness of reflexive self-description in reports. It may not be possible for authors to estimate the depth of description desired by reviewers without guidance.

Participants or Other Data Sources

- Provide the numbers of participants/documents/events analyzed.
- Describe the demographics/cultural information, perspectives of participants, or characteristics of data sources that might influence the data collected.
- Describe existing data sources, if relevant (e.g., newspapers, internet, archive).
- Provide data repository information for openly shared data, if applicable.
- Describe archival searches or process of locating data for analyses, if applicable.

Researcher–Participant Relationship

- Describe the relationships and interactions between researchers and participants relevant to the research process and any impact on the research process (e.g., was there a relationship prior to research, are there any ethical considerations relevant to prior relationships).

Participant Recruitment

Recruitment Process

- Describe the recruitment process (e.g., face-to-face, telephone, mail, email) and any recruitment protocols.
- Describe any incentives or compensation, and provide assurance of relevant ethical processes of data collection and consent process as relevant (may include institutional review board approval, particular adaptations for vulnerable populations, safety monitoring).
- Describe the process by which the number of participants was determined in relation to the study design.
- Provide any changes in numbers through attrition and final number of participants/sources (if relevant, refusal rates or reasons for dropout).
- Describe the rationale for decision to halt data collection (e.g., saturation).
- Convey the study purpose as portrayed to participants, if different from the purpose stated.

Recruitment Process (*continued*)

Guidance for Authors/Reviewers

- The order of the recruitment process and the selection process and their contents may be determined in relation to the authors' methodological approach. Some authors will determine a selection process and then develop a recruitment method based on those criteria. Other authors will develop a recruitment process and then select participants responsively in relation to evolving findings.

Guidance for Reviewers

- There is no agreed-upon minimum number of participants for a qualitative study. Rather, the author should provide a rationale for the number of participants chosen.

Participant Selection

- Describe the participants/data source selection process (e.g., purposive sampling methods, such as maximum variation; convenience sampling methods, such as snowball selection; theoretical sampling; diversity sampling) and inclusion/exclusion criteria.
- Provide the general context for the study (when data were collected, sites of data collection).
- If your participant selection is from an archived data set, describe the recruitment and selection process from that data set as well as any decisions in selecting sets of participants from that data set.

Guidance for Authors

- A statement can clarify how the number of participants fits with practices in the design at hand, recognizing that transferability of findings in qualitative research to other contexts is based in developing deep and contextualized understandings that can be applied by readers rather than quantitative estimates of error and generalizations to populations.

Guidance for Authors/Reviewers

- The order of the recruitment process and the selection process and their contents may be determined in relation to the authors' methodological approach. Some authors will determine a selection process and then develop a recruitment method based on those criteria. Other authors will develop a recruitment process and then select participants responsively in relation to evolving findings.

Data Collection

Data Collection/Identification Procedures

- State the form of data collected (e.g., interviews, questionnaires, media, observation).
- Describe the origins or evolution of the data-collection protocol.
- Describe any alterations of data-collection strategy in response to the evolving findings or the study rationale.
- Describe the data-selection or data-collection process (e.g., were others present when data were collected, number of times data were collected, duration of collection, context).
- Convey the extensiveness of engagement (e.g., depth of engagement, time intensiveness of data collection).
- For interview and written studies, indicate the mean and range of the time duration in the data-collection process (e.g., interviews were held for 75 to 110 min, with an average interview time of 90 min).
- Describe the management or use of reflexivity in the data-collection process, as it illuminates the study.
- Describe questions asked in data collection: content of central questions, form of questions (e.g., open vs. closed).

© 2024 American Psychological Association

Data Collection/Identification Procedures *(continued)*

Guidance for Reviewers

- Researchers may use terms for data collection that are coherent within their research approach and process, such as "data identification," "data collection," or "data selection." Descriptions should be provided, however, in accessible terms in relation to the readership.
- It may not be useful for researchers to reproduce all of the questions they asked in an interview, especially in the case of unstructured or semistructured interviews as questions are adapted to the content of each interview.

Recording and Data Transformation

- Identify data audio/visual recording methods, field notes, or transcription processes used.

Analysis

Data-Analytic Strategies

- Describe the methods and procedures used and for what purpose/goal.
- Explicate in detail the process of analysis, including some discussion of the procedures (e.g., coding, thematic analysis) following a principle of transparency.
- Describe coders or analysts and their training, if not already described in the researcher description section (e.g., coder selection, collaboration groups).
- Identify whether coding categories emerged from the analyses or were developed a priori.
- Identify units of analysis (e.g., entire transcript, unit, text) and how units were formed, if applicable.
- Describe the process of arriving at an analytic scheme, if applicable (e.g., if one was developed before or during the analysis or was emergent throughout).
- Provide illustrations and descriptions of the analytic scheme development, if relevant.
- Indicate software, if used.

Guidance for Authors

- Provide rationales to illuminate analytic choices in relation to the study goals.

Guidance for Reviewers

- Researchers may use terms for data analysis that are coherent within their research approach and process (e.g., "interpretation," "unitization," "eidetic analysis," "coding"). Descriptions should be provided, however, in accessible terms in relation to the readership.

Methodological Integrity

- Demonstrate that the claims made from the analysis are warranted and have produced findings with methodological integrity. The procedures that support methodological integrity (i.e., fidelity and utility) typically are described across the relevant sections of a paper, but they could be addressed in a separate section when elaboration or emphasis would be helpful. Issues of methodological integrity include the following:
  - Assess the *adequacy* of the data in terms of its ability to capture forms of diversity most relevant to the question, research goals, and inquiry approach.
  - Describe how the *researchers' perspectives* were managed in both the data collection and analysis (e.g., to limit their effect on the data collection, to structure the analysis).

Methodological Integrity *(continued)*

  - Demonstrate that findings are *grounded* in the evidence (e.g., using quotes, excerpts, or descriptions of researchers' engagement in data collection).
  - Demonstrate that the contributions are *insightful* and *meaningful* (e.g., in relation to the current literature and the study goal).
  - Provide relevant *contextual* information for findings (e.g., setting of study, information about participants, interview question asked is presented before excerpt as needed).
  - Present findings in a *coherent* manner that makes sense of contradictions or disconfirming evidence in the data (e.g., reconcile discrepancies, describe why a conflict might exist in the findings).
- Demonstrate *consistency* with regard to the analytic processes (e.g., analysts may use demonstrations of analyses to support consistency, describe their development of a stable perspective, interrater reliability, consensus) or describe responses to inconsistencies, as relevant (e.g., coders switching midway through analysis, an interruption in the analytic process). If alterations in methodological integrity were made for ethical reasons, explicate those reasons and the adjustments made.
- Describe how support for claims was supplemented by any checks added to the qualitative analysis. Examples of supplemental checks that can strengthen the research may include
  - transcripts/data collected returned to participants for feedback
  - triangulation across multiple sources of information, findings, or investigators
  - checks on the interview thoroughness or interviewer demands
  - consensus or auditing process
  - member checks or participant feedback on findings
  - data displays/matrices
  - in-depth thick description, case examples, or illustrations
  - structured methods of researcher reflexivity (e.g., sending memos, field notes, diary, logbooks, journals, bracketing)
  - checks on the utility of findings in responding to the study problem (e.g., an evaluation of whether a solution worked)

Guidance for Reviewers:

- Research does not need to use all or any of the checks (as rigor is centrally based in the iterative process of qualitative analyses, which inherently includes checks within the evolving, self-correcting iterative analyses), but their use can augment a study's methodological integrity. Approaches to inquiry have different traditions in terms of using checks and which checks are most valued.

Findings/Results

Findings/Results Subsections

- Describe research findings (e.g., themes, categories, narratives) and the meaning and understandings that the researcher has derived from the data analysis.
- Demonstrate the analytic process of reaching findings (e.g., quotes, excerpts of data).
- Present research findings in a way that is compatible with the study design.
- Present synthesizing illustrations (e.g., diagrams, tables, models), if useful in organizing and conveying findings. Photographs or links to videos can be used.

© 2024 American Psychological Association

Findings/Results Subsections *(continued)*

Guidance for Authors

- Findings presented in an artistic manner (e.g., a link to a dramatic presentation of findings) should also include information in the reporting standards to support the research presentation.
- Use quotes or excerpts to augment data description (e.g., thick, evocative description, field notes, text excerpts), but these should not replace the description of the findings of the analysis.

Guidance for Reviewers

- The findings section tends to be longer than in quantitative papers because of the demonstrative rhetoric needed to permit the evaluation of the analytic procedure.
- Depending on the approach to inquiry, findings and discussion may be combined or a personalized discursive style might be used to portray the researchers' involvement in the analysis.
- Findings may or may not include quantified information, depending upon the study's goals, approach to inquiry, and study characteristics.

Discussion

Discussion Subsections

- Describe the central contributions and their significance in advancing disciplinary understandings.
- Describe the types of contributions made by findings (e.g., challenging, elaborating on, and supporting prior research or theory in the literature describing the relevance) and how findings can be best utilized.
- Identify similarities and differences from prior theories and research findings.
- Reflect on any alternative explanations of the findings.
- Identify the study's strengths and limitations (e.g., consider how the quality, source, or types of the data or the analytic processes might support or weaken its methodological integrity).
- Describe the limits of the scope of transferability (e.g., what should readers bear in mind when using findings across contexts).
- Revisit any ethical dilemmas or challenges that were encountered, and provide related suggestions for future researchers.
- Consider the implications for future research, policy, or practice.

Guidance for Reviewers

- Accounts could lead to multiple solutions rather than a single one. Many qualitative approaches hold that there may be more than one valid and useful set of findings from a given data set.

© 2024 American Psychological Association
